# The influence of descriptive norms and media trust on male HPV vaccination willingness: an extended protection motivation theory approach

**DOI:** 10.3389/fpsyg.2025.1640345

**Published:** 2025-11-21

**Authors:** Zhuliu Gong, Yi Guo

**Affiliations:** School of Journalism and Communication, Chongqing University, Chongqing, China

**Keywords:** HPV vaccine, males, protection motivation theory, descriptive norms, media trust

## Abstract

**Purpose:**

Human papillomavirus (HPV) poses a substantial health risk to men, yet male vaccination remains under-prioritized in China. This study examines how five Protection Motivation Theory (PMT) cognitions (perceived severity, perceived vulnerability, response efficacy, self-efficacy, and response cost), together with descriptive norms and media trust, shape Chinese men’s willingness to receive the HPV vaccine. We further assess both direct effects and indirect (mediated) pathways linking these factors to vaccination willingness.

**Participants and methods:**

A nationwide online quota survey collected 3,013 valid responses from men aged 18–45 across 120 Chinese cities. Five-point Likert scales assessed perceived severity, perceived vulnerability, response efficacy, self-efficacy, and response cost (Protection Motivation Theory constructs), as well as descriptive norms, media trust, and HPV vaccination willingness. Structural equation modeling (AMOS 29.0) estimated direct paths while controlling for age, education, income, and marital status as covariates, and bias-corrected bootstrapping was used to test indirect (mediated) effects.

**Results:**

Vaccination willingness was positively associated with perceived severity (*β* = 0.307, 95% CI [0.241, 0.372]), perceived vulnerability (*β* = 0.175, 95% CI [0.052, 0.303]), response efficacy (*β* = 0.297, 95% CI [0.228, 0.357]), self-efficacy (*β* = 0.133, 95% CI [0.041, 0.215]), descriptive norms (*β* = 0.183, 95% CI [0.126, 0.239]), and media trust (*β* = 0.133, 95% CI [0.052, 0.251]) (all *p* < 0.01). Perceived response cost was negatively associated with willingness (*β* = −0.314, 95% CI [−0.372, −0.246], *p* < 0.001). Descriptive norms indirectly promoted willingness via perceived severity and response efficacy; media trust exerted three indirect paths through perceived severity, perceived vulnerability, and response efficacy.

**Conclusion:**

Men’s cognitive appraisal of HPV risks and their coping efficacy underpin vaccination intentions. Social cues (descriptive norms) and reliable information sources (media trust) reinforce these cognitions, amplifying willingness both directly and through PMT constructs. Interventions should normalise male vaccination, enhance message credibility, and alleviate financial or procedural burdens to broaden HPV vaccine uptake among men.

## Introduction

Human Papillomavirus (HPV) infection ranks among the most common sexually transmitted diseases worldwide, with virtually every sexually active individual likely to contract HPV at some point during their lifetime (World Health Organization, 2017). According to statistical data from the World Health Organization (WHO), HPV infections constitute 15–20% of globally transmitted sexual diseases, with approximately 630,000 new cancer cases annually linked to HPV infection (Zou et al., 2022). The International Agency for Research on Cancer (IARC) reports that roughly 58.4% of new cervical cancer cases are concentrated in Asia, with China alone accounting for about 18% of these cases (Singh et al., 2023). HPV not only serves as the primary causative agent for cervical cancer in women, but epidemiological studies also indicate that HPV infection can lead to genital warts, intraepithelial neoplasia, and invasive cancers in men (World Health Organization, 2024; Staadegaard et al., 2022). Consequently, HPV infection has emerged as a significant public health challenge globally, particularly in developing nations (Patel et al., 2018).

Currently, HPV vaccines have demonstrated remarkable safety and efficacy in preventing HPV-related diseases, including cervical and genital cancers. Evidence-based medical research confirms that increasing HPV vaccination coverage can significantly reduce the incidence of cervical cancer in women and genital cancers in men (Garland et al., 2016; Markowitz et al., 2016). China lags approximately ten years behind most developed countries in promoting HPV vaccination (Pan et al., 2016). Although data indicates that HPV vaccine batch approval volumes have shown significant growth, becoming a core category in the non-mandatory vaccination market (Wang, 2021), the current vaccination coverage remains extremely low due to HPV vaccines not yet being included in China’s National Immunization Program (NIP) (Deng et al., 2021). Vaccination rates among adolescents are below 3%, while the overall population vaccination rate remains under 6% (C-CPCoGPM A, 2021). Furthermore, males were previously excluded from HPV vaccination programs until January 18th, when Merck held press conferences in Beijing, Shanghai, and Guangzhou to announce the official launch of the quadrivalent HPV vaccine for males in China.

There is growing recognition that vaccinating boys not only effectively reduces the incidence of human papillomavirus infections and related diseases in male populations, but also significantly diminishes passive infection risks among females by disrupting the transmission chain, thus creating a synergistic effect of herd immunity protection (Georgousakis et al., 2012; Kim et al., 2021). This gender-linked prevention mechanism has emerged as a crucial direction for optimizing HPV prevention strategies (Fu et al., 2024). Nevertheless, existing research still predominantly focuses on women’s willingness and behaviors regarding HPV vaccination (Zhang Z. et al., 2023). This gendered medical practice further reinforces the public perception stereotype of “male non-vulnerability” resulting in significant knowledge gaps about HPV vaccines among male populations (Varer Akpinar and Alanya Tosun, 2023; Xie et al., 2023). Limited knowledge about HPV, low risk perception, and vaccine costs —which are a primary deterrent given that the multi-dose series is not included in China’s National Immunization Program (NIP) and can cost upwards of 2,000–4,000 RMB (approx. 300–600 USD) for the full series— have led to relatively low willingness and rates of HPV vaccination among males (Chen et al., 2021; Liu et al., 2023; Yao et al., 2022; Zhang S. et al., 2023). Given these circumstances, developing pathways to enhance health literacy and promote vaccination behaviors among age-appropriate males has become a priority issue for interdisciplinary health communication research and a core concern for public health decision-making.

Health behavior change theories provide multidimensional frameworks for analyzing HPV vaccination decision-making mechanisms, with different theoretical perspectives revealing various pathways through which individual cognition and external environments influence vaccination willingness (Prestwich et al., 2015). The Protection Motivation Theory has been widely applied in vaccination research due to its systematic explanation of how threat assessment (perceived severity and vulnerability) and coping assessment (response efficacy and self-efficacy) drive health decisions (Alwreikat, 2024; Fan et al., 2025; Luo et al., 2024; Okuhara et al., 2022; Tong et al., 2021). According to PMT, individuals’ protective behaviors are motivated by two appraisal processes: threat appraisal and coping appraisal. Threat appraisal encompasses perceived severity (beliefs about the seriousness of the health threat) and perceived vulnerability (beliefs about one’s vulnerability to the threat). Coping appraisal comprises response efficacy (beliefs about the effectiveness of the recommended action) and self-efficacy (confidence in one’s ability to perform the action). The net motivation is also weighed against response costs (perceived barriers to performing the behavior). A Canadian study examining vaccination willingness across different HPV vaccine target populations found that response efficacy correlates with positive vaccination willingness, while perceived vulnerability alone cannot serve as an independent predictor of vaccination willingness (Liu et al., 2016). A study from China further confirmed PMT’s effectiveness in predicting factors influencing vaccine uptake. Except for response costs, other PMT variables significantly influenced HPV vaccination willingness among male groups through direct or indirect pathways. Knowledge levels and perceived severity indirectly affected vaccination willingness through chain mediation effects, while perceived vulnerability, response efficacy, and self-efficacy directly predicted vaccination willingness (C-CPCoGPM A, 2021).

Protection Motivation Theory (PMT) offers a systematic account of how threat appraisal (perceived severity and perceived vulnerability) and coping appraisal (response efficacy and self-efficacy) motivate preventive action. Yet classic critiques argue that PMT, in its canonical form, under-specifies social-contextual forces—notably the role of social norms in shaping intentions and behavior (Fishbein, 2007). Building on this insight, we extend PMT by incorporating two contextual determinants that are theoretically poised to inform PMT cognitions and downstream intentions: descriptive norms and media trust.

Following Rimal and Real, social norms are group-level expectations that guide behavior (Rimal and Real, 2003); a useful distinction is between descriptive norms (what most people do) and injunctive norms (what most people approve) (Cialdini et al., 1990). In vaccination contexts, injunctive cues embedded in public policy may face efficacy constraints under conditions of institutional distrust, whereas descriptive norms can cultivate a perceived “normalcy of vaccination” through social observation, thereby motivating uptake (Jaffe et al., 2022). Empirical work documents robust links between descriptive norms and HPV vaccination intentions across settings—including among men (Peterson et al., 2022; Xiao and Borah, 2021; Yarmohammadi et al., 2023). Moreover, norm exposure can shape risk perception and efficacy beliefs, which in turn influence willingness (Stout et al., 2020; Wang et al., 2023). In the present framework, descriptive norms are expected to directly promote vaccination willingness and indirectly do so by reinforcing PMT cognitions.

Media function as central infrastructures for risk information acquisition and social interaction; accordingly, individuals’ exposure to and trust in these sources shape information processing and health behavior (Li and Bautista, 2020; Lin and Lagoe, 2013). Because lay audiences often cannot directly verify complex scientific claims, trust in knowledge authorities becomes a necessary cognitive shortcut for attitude formation (Blöbaum, 2016). Consequently, information sources with higher credibility often have more pronounced effects on attitude changes (Petty and Cacioppo, 1984), and people filter information according to their trust in media, with varying degrees of trust in different information channels determining different behavioral intentions (Zhao et al., 2020). In vaccination contexts, higher media trust is consistently associated with stronger vaccination willingness (Chen et al., 2023; Glauberman et al., 2024; Limbu and Gautam, 2023; Pérez et al., 2016). Beyond direct effects, media trust has cognitive and affective impacts (Ball-Rokeach and DeFleur, 1976). And may operate through PMT variables—e.g., amplifying perceived severity and vulnerability and reinforcing response efficacy—to shape motivation (Li and Sun, 2021). Within our extension, media trust therefore serves as a contextual driver that both directly promotes willingness and indirectly strengthens PMT cognitions.

Based on the above, this study proposes the following research hypotheses:

*H1:* (a) Perceived severity, (b) perceived vulnerability, (c) response efficacy, (d) self-efficacy, (e) descriptive norms, and (f) media trust positively correlate with male HPV vaccination willingness; (g) response cost negatively correlates with male HPV vaccination willingness.

*H2:* (a) Perceived severity, (b) perceived vulnerability, (c) self-efficacy, and (d) response efficacy will mediate the relationship between descriptive norms and men' s HPV vaccination willingness.

*H3:* (a) Perceived severity, (b) perceived vulnerability, (c) self-efficacy, and (d) response efficacy will mediate the relationship between media trust and men' s HPV vaccination willingness.

**Figure 1:**
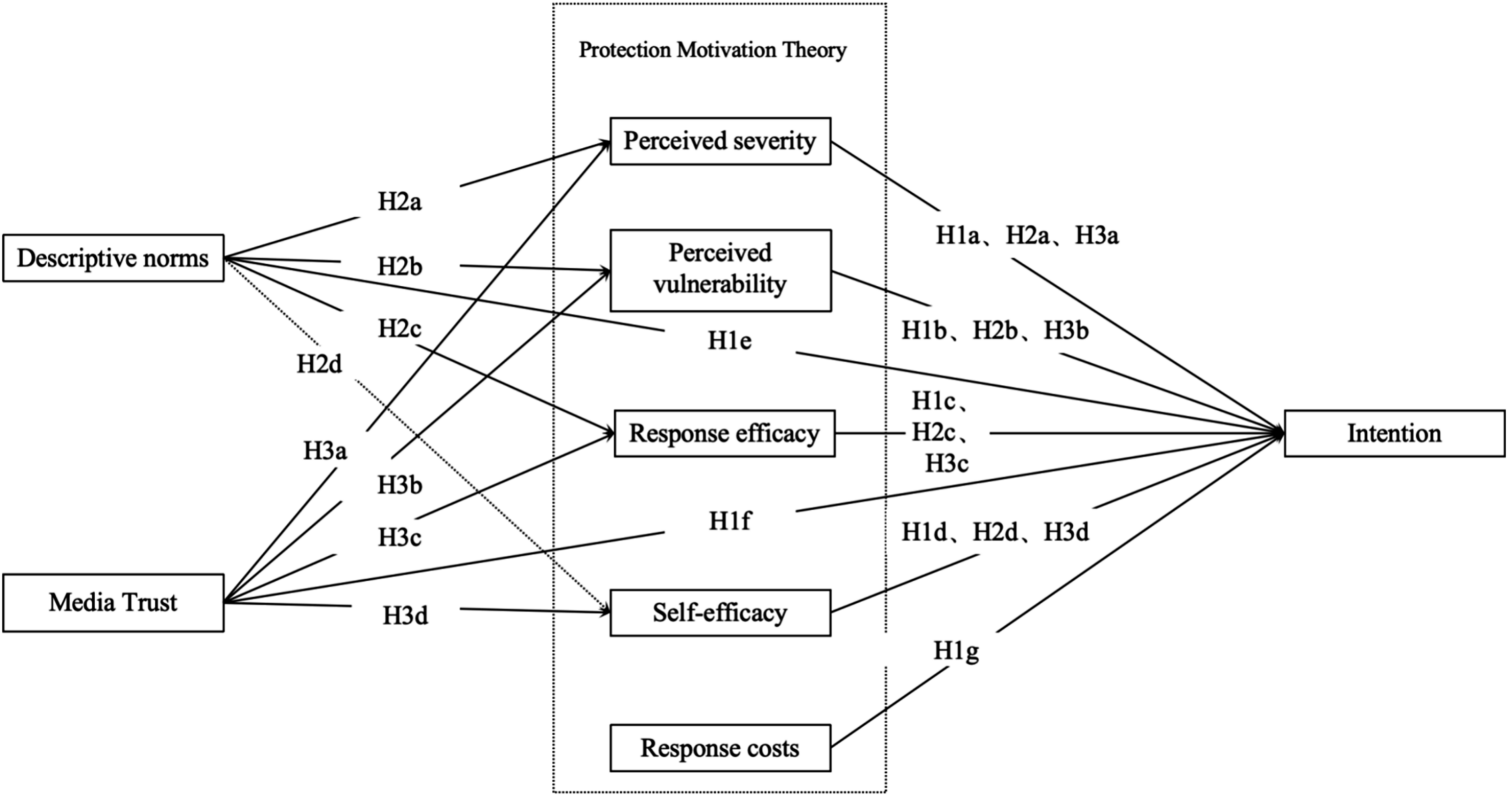
Hypothesized structural model.

Based on the theoretical integration above, this study proposes a comprehensive conceptual framework (see Figure 1).

## Materials and methods

### Survey methods

This study employed a multi-stage sampling design with quota sampling at the final stage across 120 cities in mainland China (23 provinces, 5 autonomous regions, and all 4 municipalities). Between March 15 and April 1, 2025, we first selected 120 survey sites: all provincial capitals, all four municipalities, and 2–6 randomly selected prefecture-level cities within each province and autonomous region. Within each selected city, trained local survey administrators recruited eligible men aged 18–45 and supervised on-site completion of the online questionnaire. Quota targets were used within cities to approximate demographic diversity. A total of 3,200 questionnaires were distributed and 3,013 valid responses were obtained, yielding an effective response rate of 94.16%.

Each sample city engaged at least one survey administrator or team for data collection. Individual administrators were responsible for collecting 30–90 questionnaires, while survey teams handled 100–200 questionnaires. Administrators conducted one-on-one, face-to-face questionnaire distribution through the “WJX.cn” platform.^1^ Respondents completed the questionnaire by accessing a unique link, with administrators ensuring informed consent and assigning a unique identifier to each submission. When respondents possessed cognitive ability but limited mobility, administrators provided one-on-one assistance and completed the questionnaire on their behalf. This study was conducted according to the guidelines of the Declaration of Helsinki, and approved by the Academic Board of the School of Journalism and Communication, Chongqing University (9 January 2025 of approval). Online informed consent was obtained from all subjects involved in the study.

### Study population

The China Comprehensive Cervical Cancer Prevention and Control Guidelines indicates that the appropriate age range for HPV vaccination is 9–45 years. We targeted men aged 18–45 because (i) the recommended vaccination window extends up to 45 years, (ii) 18 marks legal adulthood for independent consent, and (iii) this range captures sexually active men with higher exposure risk, for whom vaccination may yield both direct protection and indirect benefits via transmission interruption. Using SPSS 26.0, we analyzed the sociodemographic characteristics of the 3,013 male participants (Table 1).

**Table 1:** Sample characteristics.

Variable	Category	Frequency (*n*)	Percentage (%)
Age	18–20	158	5.2
	21–30	1,616	53.6
	31–40	1,058	35.1
	≥41	181	6.0
Marital status	Married	1885	62.6
	Unmarried	1,128	37.4
Residence (Hukou)	Urban	2,358	78.3
	Rural	655	21.7
Education	High school or below	742	24.6
	Junior college	770	25.6
	Bachelor’s	762	25.3
	Master’s and above	739	24.5
Monthly income (CNY)	≤3,000	302	10.0
	3,001–5,000	1,443	47.9
	5,001–8,000	1,026	34.1
	≥8,001	242	8.0
Smoking	Yes	732	24.3
	No	2,281	75.7
Number of sexual partners	1	2,149	71.3
	≥2	864	28.7

Inclusion criteria: (i) males aged 18–45; (ii) Chinese citizenship; (iii) permanent residents of China (time away ≤1 month per year); (iv) voluntary participation with signed informed consent; (v) ability to complete the online questionnaire independently or with administrator assistance; (vi) comprehension of the meaning expressed in each questionnaire item.

Exclusion criteria: (i) individuals with impaired consciousness or mental disorders; (ii) current participants in similar research studies; (iii) unwilling participants.

### Questionnaire design and variable measurement

#### General demographic information questionnaire

The questionnaire collected the following demographic and behavioral variables: age, marital status, household registration status, highest educational attainment, average monthly personal income (CNY), smoking status, and number of sexual partners.

#### Protection motivation questionnaire

Based on Protection Motivation Theory (PMT), this study adapted the dimensional structure and measurement methods from the Breast Cancer PMT Scale (Zhang et al., 2021), modified for cultural appropriateness and gender specificity for male HPV vaccination behaviors. The revised questionnaire included five core dimensions with 16 items, rated on a five-point Likert scale (1 = “strongly disagree” to 5 = “strongly agree”). The dimensions, definitions, measurement items, and scoring methods were as follows:

Perceived vulnerability included 3 items measuring individuals’ risk perception of HPV infection. Perceived severity contained 3 items assessing individuals’ perception of potential health consequences from HPV infection. Perceived self-efficacy comprised 4 items measuring individuals’ confidence in completing HPV vaccination. Response efficacy included 3 items evaluating individuals’ acknowledgment of HPV vaccination effectiveness. The response cost contained 3 items measuring perceived barriers to HPV vaccination in terms of economic, psychological, and time costs. Each PMT subscale score was computed as the mean of its items (1 = strongly disagree to 5 = strongly agree). Higher values indicate stronger perceived severity/vulnerability/efficacy or higher perceived cost. Example items include: severity—"HPV infection may cause serious illness”; vulnerability—"I am likely to be exposed to HPV in my lifetime”; response efficacy—"HPV vaccination can effectively prevent HPV infection”; self-efficacy—"I can complete all required vaccine doses on schedule”; response cost—"The vaccine is too expensive or time-consuming for me”.

#### Descriptive norms scale

Descriptive norms were measured using items adapted from Liu et al. (2024), which assess the perceived prevalence of protective behavior among salient others. The revised scale contained four items rated on a five-point Likert scale (1 = “strongly disagree” to 5 = “strongly agree”). Higher scores indicate stronger perceived normative support for HPV vaccination among male peers and close contacts. Items included: “Most of my male friends are willing to get HPV vaccination,” “Among my male colleagues or classmates, most plan to get HPV vaccination in the future,” “My close male family members (e.g., brothers, father) support and consider getting HPV vaccination,” and “Most male members in my social circle (e.g., close friends, partners) consider HPV vaccination a necessary health choice”.

#### Media trust scale

This study developed a media trust scale for the male HPV vaccination context by integrating Li and Sun’s (2021) “traditional-social-interpersonal” three-dimensional media trust scale and Zhou et al.’s (2023) “official-social” two-dimensional media trust scale. The scale comprised 10 items covering official/traditional media (national and local television broadcasts, government websites), social media (official new media accounts and health-related self-media accounts), and interpersonal communication (WeChat groups, verbal exchanges with relatives and friends). Respondents rated their trust in information from these sources over the past month using a 5-point Likert scale (1 = “very untrustworthy” to 5 = “very trustworthy”); higher scores indicated stronger overall media trust.

#### Male HPV vaccination willingness scale

Based on Fishbein et al.’s scale (Fishbein, 2008), this study developed a contextualized 4-item “vaccination willingness” scale addressing both “free vaccination scenarios” and “self-funded vaccination scenarios costing no less than 2,000 yuan.” Respondents self-reported their vaccination likelihood using a 5-point Likert scale (1 = “extremely unlikely” to 5 = “extremely likely”). While willingness may differ between free and self-funded scenarios, the core construct measured was the general psychological intention to vaccinate. A preliminary Principal Component Analysis (PCA) confirmed that all four items loaded strongly onto a single factor. More importantly, the scale showed high internal consistency (Cronbach’s *α* = 0.873). This suggests that despite the cost difference, the items collectively tap into a unidimensional construct of “general vaccination willingness.” Therefore, the average score of the four items was used in the final model to represent this latent variable.

### Statistical analysis

Statistical Analysis. Descriptive statistics, reliability, and validity checks were conducted in SPSS 26.0. Structural equation modeling (SEM) was conducted in AMOS 29.0 with standardized variables. Age, education, income, and marital status were included as controls in all structural models. Model fit was evaluated using CMIN/DF, RMSEA, GFI, TLI, IFI, and CFI. Indirect effects were tested with bias-corrected bootstrapping (5,000 resamples, 95% CIs). Multicollinearity was assessed via variance inflation factors (VIF < 5).

Item-level missing data were minimal (all variables <3%). Missing responses in the structural equation modeling were handled using full information maximum likelihood (FIML) in AMOS 29.0, which allows all available cases to be retained under a missing-at-random assumption.

## Results

### Suitability assessment

To assess the underlying factor structure of all measurement items, a principal component analysis (PCA) was conducted on the 34 items comprising the eight latent constructs. Prior to extraction, sampling adequacy was verified. The Kaiser–Meyer–Olkin (KMO) measure of sampling adequacy was 0.928, and Bartlett’s test of sphericity yielded a χ^2^ value of 48,913.678 (df = 561, *p* < 0.001). These statistics indicate that the data are well-suited for PCA analysis (Table 2).

**Table 2:** KMO and Bartlett’s test of Sphericity.

KMO measure of sampling adequacy		0.928
Bartlett’s test of Sphericity	Approx. Chi-square	48913.678
	DF	561
	SIG.	<0.001

### Principal component analysis

PCA extracted eight principal components that together explained 66.335% of the total variance—exceeding the 60% benchmark commonly applied in behavioral science. In the unrotated solution, the first component accounted for 27.415% of the variance, which is below the 40% threshold suggested for detecting common-method bias; hence, common-method bias is unlikely to compromise further analyses (Table 3).

**Table 3:** Total variance explained.

Component	Initial eigenvalues			Sums of squared loadings after extraction			Sums of squared loadings after rotation		
	Total	% of variance	Cumulative %	Total	% of variance	Cumulative %	Total	% of variance	Cumulative %
1	9.321	27.415	27.415	9.321	27.415	27.415	6.346	18.663	18.663
2	4.018	11.816	39.231	4.018	11.816	39.231	2.819	8.292	26.955
3	2.186	6.431	45.662	2.186	6.431	45.662	2.692	7.918	34.873
4	1.804	5.307	50.969	1.804	5.307	50.969	2.600	7.646	42.52
5	1.624	4.777	55.746	1.624	4.777	55.746	2.313	6.803	49.322
6	1.261	3.709	59.455	1.261	3.709	59.455	1.967	5.785	55.107
7	1.236	3.634	63.089	1.236	3.634	63.089	1.926	5.663	60.771
8	1.103	3.245	66.335	1.103	3.245	66.335	1.892	5.564	66.335

### Reliability and validity assessment

Cronbach’s *α* coefficients for the eight latent constructs span 0.713 (Perceived Severity) to 0.931 (Media Trust), exceeding the conventional 0.70 threshold and confirming strong internal consistency across all scales. Confirmatory factor analysis shows that standardised factor loadings range from 0.715 to 0.850, well above the 0.50 criterion; average variance extracted (AVE) values fall between 0.540 and 0.694; and composite reliability (CR) indices range from 0.779 to 0.935. Together, these indicators demonstrate satisfactory convergent validity and reliability.

Discriminant validity, assessed via the Fornell–Larcker criterion, is likewise adequate: for every construct, the square root of its AVE (0.735–0.833) surpasses all corresponding inter-construct correlations reported in Table 4, indicating that each construct is empirically distinct. Collectively, the measurement model therefore exhibits robust internal consistency, convergent validity, and discriminant validity, providing a sound basis for subsequent structural modelling.

**Table 4:** Convergent and discriminant validity statistics.

Construct	Estimate	AVE	CR	AVE square root	Cronbach’s *α*
PS1 ← Perceived severity	0.733	0.547	0.784	0.740	0.713
PS1 ← Perceived severity	0.733	0.547	0.784	0.740	0.713
PS2 ← Perceived severity	0.757				
PS3 ← Perceived severity	0.729				
PV1 ← Perceived vulnerability	0.741	0.568	0.798	0.754	0.737
PV2 ← Perceived vulnerability	0.741				
PV3 ← Perceived vulnerability	0.779				
SE1 ← Self-efficacy	0.813	0.640	0.877	0.800	0.848
SE2 ← Self-efficacy	0.801				
SE3 ← Self-efficacy	0.785				
SE4 ← Self-efficacy	0.800				
RE1 ← Response efficacy	0.715	0.540	0.779	0.735	0.728
RE2 ← Response efficacy	0.722				
RE3 ← Response efficacy	0.767				
RC1 ← Response costs	0.850	0.694	0.872	0.833	0.857
RC2 ← Response costs	0.815				
RC3 ← Response costs	0.833				
DN1 ← Descriptive norms	0.735	0.572	0.842	0.756	0.802
DN2 ← Descriptive norms	0.760				
DN3 ← Descriptive norms	0.746				
DN4 ← Descriptive norms	0.783				
CM1 ← Media trust	0.764	0.591	0.935	0.769	0.931
CM2 ← Media trust	0.769				
CM3 ← Media trust	0.750				
CM4 ← Media trust	0.761				
CM5 ← Media trust	0.777				
CM6 ← Media trust	0.798				
CM7 ← Media trust	0.791				
CM8 ← Media trust	0.793				
CM9 ← Media trust	0.741				
CM10 ← Media trust	0.743				
VW1 ← Vaccination Willingness	0.786	0.579	0.846	0.761	0.873
VW2 ← Vaccination Willingness	0.730				
VW3 ← Vaccination Willingness	0.737				
VW4 ← Vaccination Willingness	0.790				

### Main-effect testing

The proposed structural equation model was evaluated in AMOS 29.0. After model refinement, the fit statistics met recommended thresholds (Table 5). CMIN/DF was 4.927 (between 3 and 5 indicates adequate fit), RMSEA was 0.036 (< 0.05 denotes excellent fit), and incremental indices (GFI = 0.955, TLI = 0.955, IFI = 0.961, CFI = 0.961) all exceeded the 0.90 criterion. Collectively, these indices provide strong evidence that the structural model reproduces the observed covariance matrix with satisfactory fidelity.

**Table 5:** Model fit indices.

Index	Recommended threshold	Observed value
CMIN/DF	1–3 = excellent; 3–5 = acceptable	4.927
RMSEA	< 0.05 = excellent; < 0.08 = acceptable	0.036
GFI	> 0.90 = excellent; > 0.80 = acceptable	0.955
TLI	> 0.90 = excellent; > 0.80 = acceptable	0.955
IFI	> 0.90 = excellent; > 0.80 = acceptable	0.961
CFI	> 0.90 = excellent; > 0.80 = acceptable	0.961

To rule out multicollinearity among the exogenous predictors, variance-inflation factors were computed using the formula VIF_j_ = 1/(1−R_j_^2^) where denotes the coefficient of determination obtained by regressing predictor j on all other predictors (Fang and Wen, 2018). All VIF values were < 5, confirming that multicollinearity is not a concern in the present analysis.

**Figure 2:**
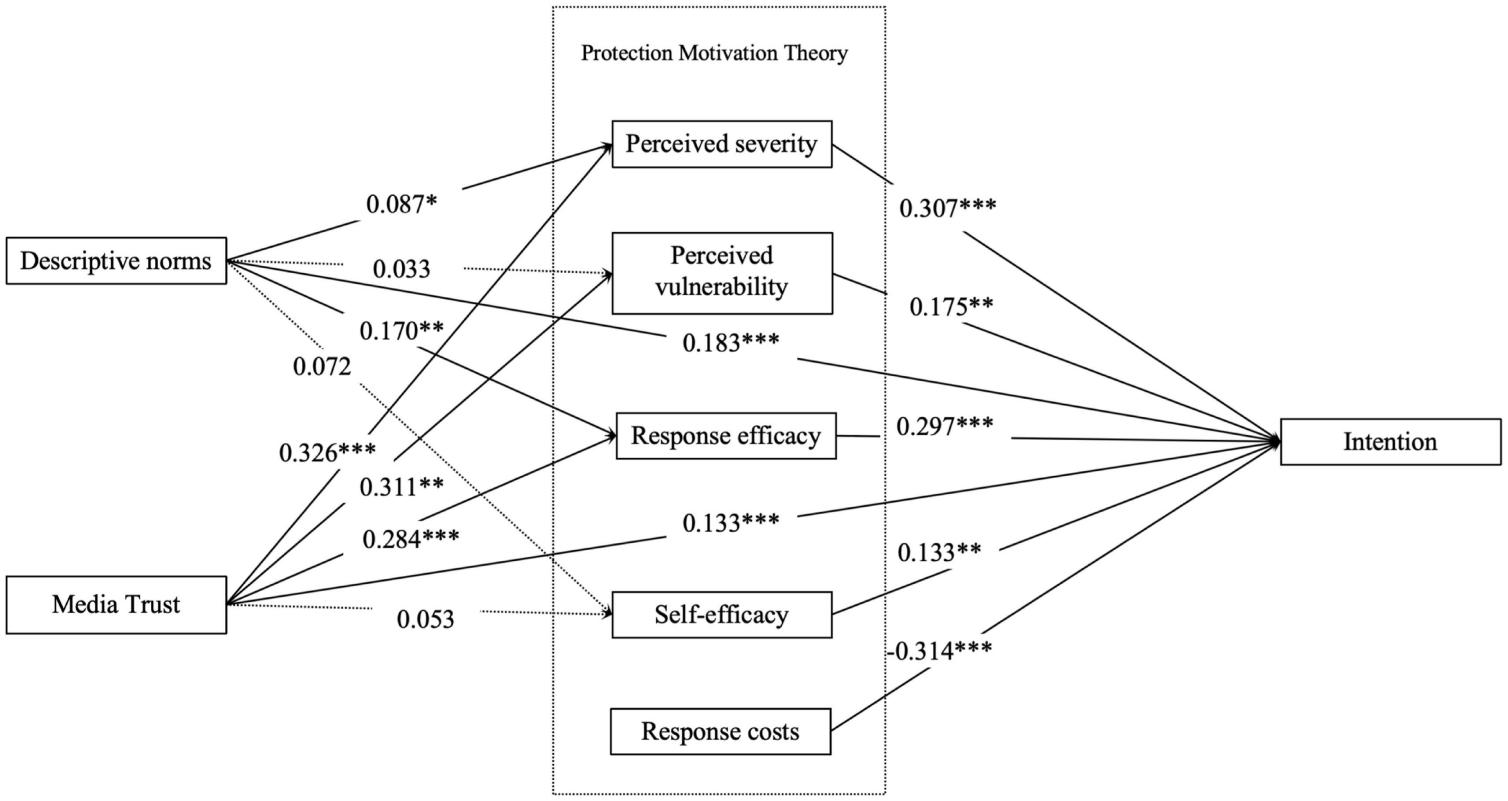
Results of path analysis. *, *p* < 0.05;**, *p* < 0.01;***, *p* < 0.001.

Figure 2 and Table 6 report standardized structural paths. The results show how sociocognitive predictors (descriptive norms, media trust) shape PMT appraisals, which in turn determine men’s HPV vaccination intention.

**Table 6:** Structural path hypothesis testing results.

Path relationship	Estimate	S.E.	C.R.	95% CI		*P*	Conclusion
				Lower	Upper		
Descriptive norms → Perceived severity	0.087	0.044	1.98	0.041	0.149	0.048	Supported
Descriptive norms → Perceived vulnerability	0.033	0.040	0.83	−0.039	0.113	0.407	Not supported
Descriptive norms → Self-efficacy	0.072	0.060	1.20	−0.019	0.173	0.230	Not supported
Descriptive norms → Response efficacy	0.170	0.053	3.20	0.051	0.291	< 0.001	Supported
Media trust → Perceived severity	0.326	0.029	11.20	0.269	0.383	< 0.001	Supported
Media trust → Perceived vulnerability	0.311	0.107	2.90	0.152	0.463	0.004	Supported
Media trust → Self-efficacy	0.053	0.044	1.20	−0.019	0.131	0.230	Not supported
Media trust → Response efficacy	0.284	0.032	9.00	0.227	0.338	< 0.001	Supported
Media trust → Vaccination intention	0.133	0.036	3.70	0.052	0.251	< 0.001	Supported
Descriptive norms → Vaccination intention	0.183	0.033	5.50	0.126	0.239	< 0.001	Supported
Perceived severity → Vaccination intention	0.307	0.034	9.00	0.241	0.372	< 0.001	Supported
Perceived vulnerability → Vaccination intention	0.175	0.062	2.80	0.052	0.303	0.005	Supported
Response efficacy → Vaccination intention	0.297	0.035	8.50	0.228	0.357	< 0.001	Supported
Self-efficacy → Vaccination intention	0.133	0.048	2.80	0.041	0.215	0.005	Supported
Response costs → Vaccination intention	−0.314	0.033	−9.50	−0.372	−0.246	< 0.001	Supported

Descriptive norms, conceptualized as the perceived prevalence of HPV vaccination among salient others, had a selective influence on PMT cognitions. Descriptive norms were positively associated with response efficacy (*β* = 0.170, 95% CI [0.051, 0.291], *p* < 0.001) and perceived severity (*β* = 0.087, 95% CI [0.041, 0.149], *p* < 0.05). By contrast, paths to perceived vulnerability (*β* = 0.033, 95% CI [−0.039, 0.113], *p* = 0.407), and self-efficacy (*β* = 0.072, 95% CI [−0.019, 0.173], *p* = 0.230) were not significant. Thus, norms in this context appear to validate “this works” more than they communicate urgency or procedural know-how. Importantly, descriptive norms also showed a direct positive association with HPV vaccination intention (*β* = 0.183, 95% CI [0.126, 0.239], *p* < 0.001), indicating that perceiving vaccination as normative among peers corresponds to greater willingness to be vaccinated (see Table 7).

**Table 7:** Hypothesis testing results.

Path	Effect	95% CI		*P*	Conclusion
		Lower	Upper		
Descriptive norms → Perceived severity → Vaccination intention	0.027	0.011	0.075	0.015	Supported
Descriptive norms → Perceived vulnerability → Vaccination intention	0.006	−0.012	0.034	0.343	Not supported
Descriptive norms → Self-efficacy → Vaccination intention	0.01	−0.004	0.038	0.404	Not supported
Descriptive norms → Response efficacy → Vaccination intention	0.05	0.012	0.105	< 0.001	Supported
Media trust → Perceived severity → Vaccination intention	0.1	0.065	0.142	0.004	Supported
Media trust → Perceived vulnerability → Vaccination intention	0.054	0.008	0.14	0.044	Supported
Media trust → Self-efficacy → Vaccination intention	0.007	−0.004	0.029	0.108	Not supported
Media trust → Response efficacy → Vaccination intention	0.084	0.053	0.124	< 0.001	Supported

Media trust, defined as confidence in the credibility and reliability of vaccine-related information sources, showed a broader pattern of influence on PMT appraisals. Higher media trust was associated with higher perceived severity of HPV (*β* = 0.326, 95% CI [0.269, 0.383], *p* < 0.001), higher perceived vulnerability (*β* = 0.311, 95% CI [0.152, 0.463], *p* < 0.05), and higher response efficacy (*β* = 0.284, 95% CI [0.227, 0.338], *p* < 0.001). The path from media trust to self-efficacy was positive in direction but not statistically significant (*β* = 0.053, 95% CI [−0.019, 0.131], *p* = 0.230). In addition, media trust showed a direct positive association with HPV vaccination intention (*β* = 0.133, 95% CI [0.052, 0.251], *p* < 0.001), suggesting that confidence in trusted information environments is linked to greater willingness to be vaccinated.

HPV vaccination intention (i.e., willingness to receive the HPV vaccine) was shaped by both threat appraisal and coping appraisal. Perceived severity (*β* = 0.307, 95% CI [0.241, 0.372], *p* < 0.001) and perceived vulnerability (*β* = 0.175, 95% CI [0.052, 0.303], *p* < 0.05) showed positive associations with intention, indicating that when men view HPV as serious and personally relevant, they are more willing to vaccinate. Coping appraisal variables were also important: response efficacy (*β* = 0.297, 95% CI [0.228, 0.357], *p* < 0.001) and self-efficacy (*β* = 0.133, 95% CI [0.041, 0.215], *p* < 0.05) were both positively associated with intention, indicating that men are more willing to vaccinate when they believe vaccination works and believe they can carry it out. Perceived response cost demonstrated a significant negative association with intention (*β* = −0.314, 95% CI [−0.372, −0.246], *p* < 0.001), underscoring cost and logistical burden as deterrents.

### Mediation analysis

Following the debate over percentile versus bias-corrected bootstrap procedures for mediation testing (Zhao et al., 2010) and drawing on the simulation evidence provided by (Chin et al., 2003), we employed the bias-corrected bootstrap routine in AMOS 29.0 with 5,000 resamples. An indirect effect was deemed significant when zero was excluded from the 95% bias-corrected confidence interval (CI).

For descriptive norms, two indirect pathways to vaccination intention emerged as statistically reliable. The indirect effect operating through perceived severity was significant (indirect effect = 0.027, 95% CI [0.011, 0.075], *p* < 0.05), indicating that perceiving HPV vaccination as common in one’s peer environment is associated with heightened perceived severity of HPV, which in turn predicts stronger intention to vaccinate. The pathway through response efficacy was also significant (indirect effect = 0.050, 95% CI [0.012, 0.105], *p* < 0.001), suggesting that descriptive norms may increase willingness to vaccinate by strengthening the belief that HPV vaccination is an effective and protective response. By contrast, the indirect pathways via perceived vulnerability (indirect effect = 0.006, 95% CI [−0.012, 0.034], *p* = 0.343) and via self-efficacy (indirect effect = 0.010, 95% CI [−0.004, 0.038], *p* = 0.404) were not statistically significant, as their confidence intervals included zero. Taken together, these findings indicate that descriptive norms motivate men’s HPV vaccination intention primarily by amplifying perceived threat severity and perceived response efficacy, rather than by elevating perceived susceptibility or one’s confidence in performing the behavior. Consistent with this pattern, H2a and H2d were supported, whereas H2b and H2c were not.

For media trust, several mediational paths to vaccination intention were significant. Greater trust in media was indirectly associated with stronger vaccination intention via perceived severity (indirect effect = 0.100, 95% CI [0.065, 0.142], *p* < 0.05) and via perceived vulnerability (indirect effect = 0.054, 95% CI [0.008, 0.140], *p* < 0.05), implying that trusted information sources heighten both perceived seriousness of HPV and perceived personal susceptibility, which in turn promote intention to vaccinate. Media trust was also indirectly related to vaccination intention through response efficacy (indirect effect = 0.084, 95% CI [0.053, 0.124], *p* < 0.001), consistent with the interpretation that credible information strengthens confidence in the effectiveness of HPV vaccination. By contrast, the pathway via self-efficacy alone was not statistically reliable (indirect effect = 0.007, 95% CI [−0.004, 0.029], *p* = 0.108). Overall, these results suggest that media trust shapes men’s HPV vaccination intention chiefly by increasing perceived threat relevance (severity and vulnerability) and confidence in the vaccine’s effectiveness, rather than by enhancing perceived behavioral capability. In line with this pattern, H3a, H3b, and H3d were supported, whereas H3c was not.

## Discussion

This study uncovered how various dimensions of Protection Motivation Theory (PMT)—including perceived severity, perceived vulnerability, response efficacy, self-efficacy, and response cost—along with extended dimensions such as descriptive norms and media trust, directly and indirectly influence male HPV vaccination willingness.

Our data reveals that male respondents exhibited low levels of both perceived severity (M = 2.375, SD = 0.913) and perceived vulnerability (M = 2.264, SD = 1.098) regarding HPV infection risk. This phenomenon can be analyzed from two perspectives: First, within the public narrative surrounding HPV vaccines, long-term feminized marketing and patriarchal discourse have symbolically framed HPV vaccines as a “girls’ vaccine,” reinforcing stereotypes of “male non-vulnerability” in public discourse while placing the primary vaccination responsibility on women (Singh et al., 2023; Varer Akpinar and Alanya Tosun, 2023). The Ripple Effect in risk perception indicates that public risk perception of crisis events diminishes in a gradient from center to periphery: the greater the distance from the risk source, the lower the subjective risk (Erdem and Swait, 2004; Kasperson et al., 1988). Consequently, we can infer that males perceive themselves as being “pushed away” from the risk center, creating a symbolic distance that leads them to subjectively become peripheral observers. Second, this asymptomatic high-exposure state may induce an optimism bias prompting males to maintain optimistic health beliefs by underestimating their personal risk. When the “female vaccine” narrative positions males on the periphery (Ripple Effect), and their asymptomatic state simultaneously fosters this optimism bias, these two mechanisms compound to further diminish male risk perception.

This study found that perceived severity and perceived vulnerability have significant positive effects on male HPV vaccination willingness, consistent with previous research findings (Fu et al., 2024; Newman et al., 2013). A study based on attribution theory also identified perceived severity and perceived vulnerability as important factors influencing HPV vaccination willingness among American college students (Vorpahl and Yang, 2018). These results support the driving role of HPV health threat perceptions in behavioral decision-making. Specifically, males’ assessment of the severity of HPV infection consequences (such as penile cancer risk and reproductive health damage) directly influences their vaccination willingness—the greater the perceived risk, the more willing they are to receive the HPV vaccine (Pask and Rawlins, 2016). This aligns with previous research conclusions among Chinese male populations (Zhou et al., 2025). However, the effect size of perceived vulnerability is relatively low, possibly stemming from the pervasive cognitive bias among males that they are not susceptible to HPV virus, which they perceive as only affecting women (Kim, 2013; McPartland et al., 2005).

The results show that response efficacy and self-efficacy have significant positive effects on male HPV vaccination willingness. Similar studies have also found that response efficacy and self-efficacy play important roles in predicting HPV vaccination willingness (Huang et al., 2021). Efficacy beliefs play a crucial role in motivating males’ willingness to engage in HPV-related protective behaviors (Pask and Rawlins, 2016). This finding is highly consistent with the core proposition of Protection Motivation Theory: when individuals evaluate health threats and coping strategies, they are more likely to develop motivation and take corresponding actions if they believe the coping behavior is effective and they are confident in implementing it (Maddux and Rogers, 1983; Rogers, 1975). In the context of this study, males are naturally more willing to be vaccinated if they are confident that HPV vaccines can effectively prevent infection (high response efficacy) and that they can successfully complete the vaccination process (high self-efficacy). This also echoes the theoretical construction of the “confidence” dimension in the Vaccine Hesitancy Model, emphasizing that vaccination confidence is a key driver of vaccination behavior (Schmid et al., 2017).

Additionally, we found that response cost negatively affects vaccination willingness, suggesting that financial expenditure, time investment, complicated procedures, or social evaluation pressures may weaken males’ willingness to receive HPV vaccines. A five-year tracking study from mainland China pointed out that difficulty in scheduling appointments and high vaccination costs significantly constrained public willingness to receive HPV vaccines (Huang et al., 2024; Okunade et al., 2017). Two studies conducted in Ethiopia and Nigeria identified the same issue (Mihretie et al., 2022). This is consistent with the cost–benefit trade-off proposed by PMT and overlaps with the “convenience/cost” dimension in the 3C model (MacDonald, 2015). In promoting HPV vaccines for males, reducing direct and indirect costs—such as providing low-cost or free vaccines, establishing campus and workplace vaccination sites, simplifying appointment processes, and ensuring barrier-free information access—can significantly increase coverage rates (Razai et al., 2021).

Beyond traditional PMT variables, this study introduced descriptive norms from social norm theory and media trust variables to further explore the influence of these social factors on male HPV vaccination willingness. Social norm theory emphasizes that individual behavior is influenced not only by personal cognition but also by the behavior of their social groups (Cialdini et al., 1991). Descriptive norms can effectively stimulate individual vaccination behavior by shaping perceptions of “vaccination normalcy.” Results indicate that descriptive norms have a significant positive effect on male HPV vaccination willingness, a finding consistent with other studies on HPV vaccination willingness, supporting the important role of descriptive norms in health behaviors (Catalano et al., 2017; Peterson et al., 2022; Wang et al., 2017). Descriptive norms drive behavioral choices by presenting empirical evidence that specific behaviors are effective. Their core mechanism provides individuals with a “decision-making shortcut”: when people perceive that a certain behavior is widely adopted, they infer, based on the logic that “the majority is correct,” that the behavior must have inherent value or practical advantages, thereby promoting emulation motivation (Cialdini et al., 1991). Descriptive norms reflect individuals’ perception of vaccination behavior in their social circles—the more they see people around them being vaccinated, the more likely they are to identify with and imitate this behavior. When individuals perceive that a behavioral norm has formed broad consensus or gained majority approval among similar groups, this reinforced group interreference effect produces irresistible influence (Xiao and Borah, 2021).

The study also found that media trust significantly positively correlates with male HPV vaccination willingness—the higher males’ trust in vaccine information sources, the stronger their willingness to be vaccinated. In other words, media trust can, to some extent, enhance males’ acceptance of HPV vaccines, thereby promoting increased vaccination willingness. This research finding is consistent with similar studies on public HPV vaccination willingness (Anandarajah et al., 2024). With the diversification of information dissemination and the proliferation of false information, individuals’ trust in media has become an important factor influencing their health decisions (Li et al., 2025). According to Osgood’s Congruity Theory, there is a balanced relationship between people (P), information sources (S), and objects (O)—the more the audience (P) trusts the information source’s (S) claims, the more consistent the information source’s (S) claims about the object (O) are with the audience’s (P) claims about the object (O), and vice versa (Werner and James, 2000). In this process, media trust can be viewed as a simplification strategy people employ when dealing with uncertain and uncontrollable complex environments (Johnson et al., 2015; Zhang et al., 2019). Furthermore, according to the Information Adoption Model (IAM), source credibility influences system users’ perception of information usefulness as a peripheral route, thereby affecting users’ intentions and behaviors (Mo et al., 2022). This finding reveals the importance of improving information transparency and credibility when promoting and disseminating HPV vaccine information to males through media, especially against the backdrop of false information and vaccine conspiracy theories. How to construct a stable relationship between “high media trust and high vaccination willingness” becomes key to increasing vaccination rates.

Through mediation effect analysis, we found that descriptive norms primarily influence vaccination willingness through perceived severity and response efficacy, while media trust produces significant mediating effects through perceived severity, perceived vulnerability, and response efficacy.

First, the influence of descriptive norms on male HPV vaccination willingness is reflected not only in direct effects but also indirectly through perceived severity and response efficacy. This is consistent with previous similar research, where scholars found that social norms can directly influence vaccination willingness and can also produce indirect effects through factors such as response efficacy (Fadda et al., 2015). Social norms, as implicit rules, profoundly govern individual decision-making and behavioral patterns, with their mechanisms originating from humans’ continuous questioning and reflection on “what constitutes appropriate behavior in specific situations (Mollen et al., 2010).” According to Stimulus-Organism-Response (S-O-R) theory, external stimuli (such as persuasive information) need to be processed internally at the organism level (including emotional and cognitive responses) before ultimately influencing behavioral intentions (Wu, 2022). Following this analytical path, we can infer that descriptive norms, as exogenous behavioral reference cues and stimuli, form mediating and moderating effects by influencing males’ perceived severity of HPV virus infection and response efficacy at cognitive and psychological levels, ultimately affecting their vaccination willingness. This result suggests that individuals’ cognition of social group vaccination behavior enhances their perception of HPV infection severity while also boosting their confidence in vaccination effectiveness. Contrary to expectations, however, self-efficacy did not play a mediating role between descriptive norms and vaccination willingness. Previous research indicates that external persuasive factors must be combined with high-intensity self-efficacy to effectively drive individuals to adopt constructive behavioral changes (Pérez et al., 2016). A cohort analysis of American college students showed that perceiving “peers commonly being vaccinated” as a descriptive norm not only directly enhances vaccination willingness but also increases vaccination willingness by improving self-efficacy similar to masculinity (Peterson et al., 2022). However, our research data does not support the above assertion, and the specific reasons warrant further exploration in future studies.

Second, media trust, as an external contextual cue, can positively shape men’s threat appraisal of HPV infection (i.e., perceived severity and perceived vulnerability) and their coping appraisal (i.e., response efficacy), thereby increasing their willingness to receive the HPV vaccine. Zhang et al. (2019) reported that public engagement with social media can strengthen trust in the information environment, which subsequently elevates perceived vulnerability and perceived severity and, through these appraisals, increases men’s willingness to be vaccinated. Our findings provide empirical evidence for the logical pathway of “trust—threat appraisal/coping appraisal—health behavior.” To our knowledge, this study is the first to reproduce this mediating effect on the issue of male HPV vaccination willingness, indicating that media trust can consistently function through PMT variables across vaccine types. According to the Influence of Presumed Media Influence (IPMI) model, the media shapes risk attitudes through both direct and indirect mechanisms: first, the direct pathway affects individual cognition through the provision of risk information; second, the indirect pathway influences individuals’ normative beliefs and preventive behaviors by shaping their perceptions of “others’ breadth of information exposure and degree of influence” and social references (Li et al., 2025). Based on the above, we can infer that media trust utilizes the second pathway as a transformative mediator through cognitive processing (control-reflective type) and psychological processing (impulsive-reflexive type), thereby influencing male HPV vaccination willingness or actual behavior.

The indirect paths from descriptive norms and media trust to vaccination willingness via self-efficacy were not significant. This suggests that, in the present context, these factors function less as action-enabling cues (i.e., increasing perceived ability to obtain the vaccine) and more as salience-enhancing cues that increase perceived severity, perceived vulnerability, and response efficacy, which did significantly mediate willingness. Culturally, HPV vaccination in China is still framed as primarily female, and male vaccination is not yet routinized; structurally, men face cost (≥1,700 CNY), access, and procedural barriers. As a result, men may recognize HPV as relevant and vaccination as effective, but still lack confidence in their ability to practically obtain the vaccine. The non-significant self-efficacy pathway therefore reflects contextual constraints rather than the irrelevance of self-efficacy as a construct.

This study’s findings, particularly regarding descriptive norms and media trust, offer significant practical implications for health communication strategies. First, interventions should shift from focusing solely on individual risk to actively ‘normalizing’ male vaccination. Campaigns using messages like “The majority of men your age are considering the HPV vaccine” could leverage descriptive norms to enhance perceived severity and response efficacy. Second, given that media trust is a critical gateway to influencing both threat and coping appraisals, public health authorities must collaborate with high-credibility media sources. This includes partnering with authoritative state media, verified medical professionals (KOLs) on social media, and interpersonal health advisors to build a trusted information ecosystem, which in turn reinforces the core cognitions that drive vaccination willingness.

## Limitations

The findings of this study should be interpreted in light of several limitations, which also provide clear avenues for future research. First, and most significantly, the study relies on a cross-sectional design. This design precludes our ability to draw firm causal conclusions. While our mediation analysis is grounded in the theoretical framework of PMT, the identified paths represent statistically significant indirect associations rather than confirmed causal chains. We must, therefore, caution against interpreting these results as definitive proof of a temporal or causal sequence. Future longitudinal or experimental studies are essential to validate the causal directionality of these relationships.

Second, our study is subject to measurement limitations. All variables were captured via self-report, which introduces the risk of common method variance (CMV) and social desirability bias. Although Harman’s single-factor test did not suggest that CMV was a pervasive issue, this test is not definitive. Furthermore, on a sensitive topic such as vaccination, responses regarding willingness may be influenced by perceived social expectations. We also measured vaccination willingness rather than actual behavior. While intention is a strong predictor, the “intention-behavior gap” is well-documented. Future research would be strengthened by incorporating objective measures, such as verified vaccination records, to complement self-reported data.

Third, our theoretical framework, while extended, has notable boundaries. We focused exclusively on descriptive norms (perceptions of what others do), omitting injunctive norms (perceptions of what others approve of). Given that both types of norms can uniquely and interactively influence health decisions, future research would benefit from testing a “dual-norm” framework to provide a more comprehensive understanding of social influence. Furthermore, our media trust scale was a composite measure combining traditional, social, and interpersonal sources. While parsimonious and reliable, this approach may conflate distinct trust mechanisms. Future work should disaggregate these sources to examine their potentially differential impacts on PMT cognitions.

Finally, our findings must be considered within the context of our sample. Our data was drawn exclusively from Chinese men aged 18–45. Consequently, the results may not be generalizable to other genders, age groups (such as adolescents, a primary target for vaccination), or cultural contexts outside of mainland China. We also acknowledge our sample has a significant urban skew (78.3%), and future studies should explicitly compare urban–rural dynamics. Moreover, although our response rate was high (94.16%) due to face-to-face administration, we did not collect data on individuals who refused to participate. This introduces a potential non-response bias, as our sample might slightly over-represent individuals who are more willing to engage with health-related topics.

## Conclusion

This study deeply analyzes the influence mechanisms of male HPV vaccination willingness by integrating Protection Motivation Theory with descriptive social norms and social trust. The main research conclusions are as follows: First, perceived severity and vulnerability cognition in threat appraisal, along with response efficacy and self-efficacy in coping appraisal, collectively constitute the fundamental driving forces of behavioral decision-making. Second, descriptive norms and media trust both directly positively influence male willingness to receive HPV vaccines and indirectly influence male HPV vaccination willingness through perceived severity, response efficacy, and perceived severity, perceived vulnerability, and response efficacy, respectively. Third, response cost has a significant negative impact on vaccination willingness.

Theoretically, this study breaks through the single-pathway health behavior explanatory model, validating an integrated framework of psychological cognitive factors interacting with social norms and media trust, providing new evidence of cross-theoretical integration for health communication research. Practically, the study proposes a three-dimensional intervention strategy: First, reduce cost barriers through policy subsidies and process optimization; second, reshape male cognition through precise risk communication and efficacy enhancement training; third, create a social atmosphere of “male vaccination normalcy” through coordinated communication via authoritative medical channels, community opinion leaders, and digital platforms.

## Data Availability

The raw data supporting the conclusions of this article will be made available by the authors, without undue reservation.

## Footnotes

^1^ https://www.wjx.cn

## References

[ref1] Alwreikat A. (2024). Vaccination hesitancy: applying the protection motivation theory on parents’ information behavior. Inf. Dev. 1:102666669241261637. doi: 10.1177/02666669241261637

[ref2] Anandarajah A. Shato T. Humble S. Barnette A. R. Brandt H. M. Klesges L. M. . (2024). The association of caregiver attitudes, information sources, and trust with HPV vaccine initiation among adolescents. Hum. Vaccin. Immunother. 20:2300879. doi: 10.1080/21645515.2023.2300879, PMID: 38174998 PMC10773709

[ref3] Ball-Rokeach S. J. DeFleur M. L. (1976). A dependency model of mass-media effects. Commun. Res. 3, 3–21. doi: 10.1177/009365027600300101

[ref4] Blöbaum B. (2016). “Trust and communication in a digitized world” in Models and concepts of trust research (Heidelberg: Springer).

[ref5] Catalano H. P. Knowlden A. P. Birch D. A. Leeper J. D. Paschal A. M. Usdan S. L. (2017). Using the theory of planned behavior to predict HPV vaccination intentions of college men. J. Am. Coll. Heal. 65, 197–207. doi: 10.1080/07448481.2016.1269771, PMID: 27960609

[ref6] C-CPCoGPM A (2021). Expert consensus on HPV vaccine application in Guangdong Province for cervical-cancer elimination. Chin. Med. J. 9, 1303–1315. doi: 10.3760/cma.j.cn431274-20210819-00911

[ref7] Chen M. M. Mott N. Clark S. J. Harper D. M. Shuman A. G. Prince M. E. . (2021). HPV vaccination among young adults in the US. JAMA 325, 1673–1674. doi: 10.1001/jama.2021.0725, PMID: 33904878 PMC8080227

[ref8] Chen S. X. Ye F. T.-f. Cheng K. L. Ng J. C. Lam B. C. Hui B. P. . (2023). Social media trust predicts lower COVID-19 vaccination rates and higher excess mortality over 2 years. PNAS Nexus 2:pgad318. doi: 10.1093/pnasnexus/pgad318, PMID: 37841324 PMC10568527

[ref9] Chin W. W. Marcolin B. L. Newsted P. R. (2003). A partial least squares latent variable modeling approach for measuring interaction effects: results from a Monte Carlo simulation study and an electronic-mail emotion/adoption study. Inf. Syst. Res. 14, 189–217. doi: 10.1287/isre.14.2.189.16018

[ref10] Cialdini R. B. Kallgren C. A. Reno R. R. (1991). A focus theory of normative conduct: a theoretical refinement and reevaluation of the role of norms in human behavior. Adv. Exp. Soc. Psychol. 24, 201–234. doi: 10.1016/S0065-2601(08)60330-5

[ref11] Cialdini R. B. Reno R. R. Kallgren C. A. (1990). A focus theory of normative conduct: recycling the concept of norms to reduce littering in public places. J. Pers. Soc. Psychol. 58, 1015–1026. doi: 10.1037/0022-3514.58.6.1015

[ref12] Deng C. Chen X. Liu Y. (2021). Human papillomavirus vaccination: coverage rate, knowledge, acceptance, and associated factors in college students in mainland China. Hum. Vaccin. Immunother. 17, 828–835. doi: 10.1080/21645515.2020.1797368, PMID: 32873128 PMC7993118

[ref13] Erdem T. Swait J. (2004). Brand credibility, brand consideration, and choice. J. Consum. Res. 31, 191–198. doi: 10.1086/383434

[ref14] Fadda M. Depping M. K. Schulz P. J. (2015). Addressing issues of vaccination literacy and psychological empowerment in the measles-mumps-rubella (MMR) vaccination decision-making: a qualitative study. BMC Public Health 15, 1–13. doi: 10.1186/s12889-015-2200-9, PMID: 26328551 PMC4556054

[ref15] Fan J. Wang Q. Deng Y. Liang J. Walker A. N. You H. (2025). Explanation of intention toward influenza vaccination among cardiovascular disease patients: an application of the extended protection motivation theory. Public Health 242, 228–235. doi: 10.1016/j.puhe.2025.03.010, PMID: 40132460

[ref16] Fang J. Wen Z.-L. (2018). Moderated mediation analysis based on structural equation modeling. J. Psychol. Sci. 41, 453–458. Availble at: https://jps.ecnu.edu.cn/EN/Y2018/V41/I2/475

[ref17] Fishbein M. (2007). Prediction and change of health behavior: applying the reasoned action approach: Psychology Press. (New Jersey: Lawrence Erlbaum Associates).

[ref18] Fishbein M. (2008). A reasoned action approach to health promotion. Med. Decis. Mak. 28, 834–844. doi: 10.1177/0272989X08326092, PMID: 19015289 PMC2603050

[ref22] Fu X. Guo X. Lu J. Zhou W. Lu Y. (2024). Acceptance of human papillomavirus vaccine among boys in Asia: a narrative review. Hum. Vaccin. Immunother. 20:2429894. doi: 10.1080/21645515.2024.2429894, PMID: 39611606 PMC11610555

[ref23] Garland S. M. Kjaer S. K. Muñoz N. Block S. L. Brown D. R. DiNubile M. J. . (2016). Impact and effectiveness of the quadrivalent human papillomavirus vaccine: a systematic review of 10 years of real-world experience. Rev. Infect. Dis. 63, 519–527. doi: 10.1093/cid/ciw354, PMID: 27230391 PMC4967609

[ref24] Georgousakis M. Jayasinghe S. Brotherton J. Gilroy N. Chiu C. Macartney K. (2012). Population-wide vaccination against human papillomavirus in adolescent boys: Australia as a case study. Lancet Infect. Dis. 12, 627–634. doi: 10.1016/S1473-3099(12)70031-2, PMID: 22445354

[ref25] Glauberman G. Liebermann E. Kornides M. L. Matsunaga M. Lim E. Zimet G. . (2024). Attitudes toward adolescent HPV vaccination after the COVID-19 pandemic: a National Survey of mothers. Vaccine 12:976. doi: 10.3390/vaccines12090976, PMID: 39340008 PMC11435469

[ref26] Huang Y. Ling J. Zhao X. Lv Q. Wang L. Wu Q. . (2024). Are HPV vaccines well accepted among parents of adolescent girls in China? Trends, obstacles, and practical implications for further interventions: a five-year follow-up study. Vaccine 12:1073. doi: 10.3390/vaccines12091073, PMID: 39340103 PMC11435455

[ref27] Huang R. Wang Z. Yuan T. Nadarzynski T. Qian H.-Z. Li P. . (2021). Using protection motivation theory to explain the intention to initiate human papillomavirus vaccination among men who have sex with men in China. Tumour Virus Res. 12:200222. doi: 10.1016/j.tvr.2021.200222, PMID: 34175495 PMC8261654

[ref28] Jaffe A. E. Graupensperger S. Blayney J. A. Duckworth J. C. Stappenbeck C. A. (2022). The role of perceived social norms in college student vaccine hesitancy: implications for COVID-19 prevention strategies. Vaccine 40, 1888–1895. doi: 10.1016/j.vaccine.2022.01.038, PMID: 35190209 PMC8789646

[ref29] Johnson F. Rowley J. Sbaffi L. (2015). Modelling trust formation in health information contexts. J. Inf. Sci. 41, 415–429. doi: 10.1177/0165551515577914

[ref30] Kasperson R. E. Renn O. Slovic P. Brown H. S. Emel J. Goble R. . (1988). The social amplification of risk: a conceptual framework. Risk Anal. 8, 177–187. doi: 10.1111/j.1539-6924.1988.tb01168.x

[ref31] Kim H. W. (2013). Gender differences in knowledge and health beliefs related to behavioral intentions to prevent human papillomavirus infection. Asia Pacific J. Public Health 25, 248–259. doi: 10.1177/1010539512444307, PMID: 22652244

[ref32] Kim D. Lee H. Kim M. (2021). Overview of human papillomavirus vaccination policy changes and its impact in the United States: lessons learned and challenges for the future. Public Health Nurs. 38, 396–405. doi: 10.1111/phn.12873, PMID: 33569854

[ref33] Li L. Bautista J. R. (2020). Incorporating communication factors in the theory of planned behavior to predict Chinese university students’ intention to consume genetically modified foods. Int. J. Commun. 14:22.

[ref34] Li Y. Gao R. Li T. Wen N. (2025). How social media amplifies HPV risk: applying the influence of presumed media influence model to the risk amplification framework. J. Risk Res. 28, 59–77. doi: 10.1080/13669877.2025.2485058, PMID: 41180919

[ref35] Li Z. Sun X. (2021). Analysis of the impact of media trust on the public’s motivation to receive future vaccinations for COVID-19 based on protection motivation theory. Vaccine 9:1401. doi: 10.3390/vaccines9121401, PMID: 34960147 PMC8708237

[ref36] Limbu Y. B. Gautam R. K. (2023). The determinants of COVID-19 vaccination intention: a meta-review. Front. Public Health 11:1162861. doi: 10.3389/fpubh.2023.1162861, PMID: 37377544 PMC10291626

[ref37] Lin C. A. Lagoe C. (2013). Effects of news media and interpersonal interactions on H1N1 risk perception and vaccination intent. Commun. Res. Rep. 30, 127–136. doi: 10.1080/08824096.2012.762907

[ref39] Liu R. W. Cheng Y. Foerster T. A. (2024). Modeling the relationship between perceived descriptive norms and willingness to practice COVID-19 preventative behaviors: a test of the mediation and moderation mechanisms in the theory of normative social behavior. Health Commun. 39, 339–351. doi: 10.1080/10410236.2023.2165257, PMID: 36628486

[ref40] Liu S. Liu K. Fu L. Liu Z. (2023). Research progress on the preventive efficacy of HPV vaccination in males. New Medicine 54, 783–786. Available at: https://kns.cnki.net/kcms2/article/abstract?v=hQtzIo_70oEC7bFvXYIX93sF7XM5Lg7Up3V7A8-LKHqS0JrH3delfVVxk9Nq8YLtL3hNU23qqCJLZNCM3sR8FDb04s6FMHxRYf7EcGBt_Mv36g2igZoXbd1o_9V3yQdAJtw_Y1qhPibD5o0897YeTF6abSEbCpIYjySsU-rMySz8ewz_TWC_rg==&uniplatform=NZKPT&language=CHS

[ref41] Liu R. Li Y. Wangen K. R. Maitland E. Nicholas S. Wang J. (2016). Analysis of hepatitis B vaccination behavior and vaccination willingness among migrant workers from rural China based on protection motivation theory. Hum. Vaccin. Immunother. 12, 1155–1163. doi: 10.1080/21645515.2015.1123358, PMID: 27078191 PMC4963072

[ref42] Luo C. He Y. Xu Y. Yang X. Wang H. (2024). How does loss-versus-gain message framing affect HPV vaccination intention? Mediating roles of discrete emotions and cognitive elaboration. Curr. Psychol. 43, 9443–9456. doi: 10.1007/s12144-023-05131-w

[ref43] MacDonald N. E. (2015). Vaccine hesitancy: definition, scope and determinants. Vaccine 33, 4161–4164. doi: 10.1016/j.vaccine.2015.04.036, PMID: 25896383

[ref44] Maddux J. E. Rogers R. W. (1983). Protection motivation and self-efficacy: a revised theory of fear appeals and attitude change. J. Exp. Soc. Psychol. 19, 469–479. doi: 10.1016/0022-1031(83)90023-9

[ref46] Markowitz L. E. Liu G. Hariri S. Steinau M. Dunne E. F. Unger E. R. (2016). Prevalence of HPV after introduction of the vaccination program in the United States. Pediatrics 137. doi: 10.1542/peds.2015-1968, PMID: 26908697

[ref47] McPartland T. S. Weaver B. A. Lee S.-K. Koutsky L. A. (2005). Men's perceptions and knowledge of human papillomavirus (HPV) infection and cervical cancer. J. Am. Coll. Heal. 53, 225–230. doi: 10.3200/JACH.53.5.225-230, PMID: 15813233

[ref48] Mihretie G. N. Liyeh T. M. Ayele A. D. Belay H. G. Yimer T. S. Miskr A. D. (2022). Knowledge and willingness of parents towards child girl HPV vaccination in Debre Tabor town, Ethiopia: a community-based cross-sectional study. Reprod. Health 19:136. doi: 10.1186/s12978-022-01444-4, PMID: 35689288 PMC9188100

[ref49] Mo M. Kuang Y. Zhu Q. Li X. Yue Q. (2022). Factors influencing users’ intention to adopt online consultation information. Modern Inform. 42, 57–68. Available at: https://kns.cnki.net/kcms2/article/abstract?v=uSrlZFhNZxKHRWQKMIPG0ADJBAN0wZxUyBA1gibPIpn_1QbdmUaSCeJ3iYJgjYlWVlkhImMPdDDeNtQRYp9iZHYp0AGZ7gXGVq53yzoKtWGrtmHllj2h_b48NlnWYzfVrKq6MKxx4M7udMPLf0Cqptk5ScFSqlbygHfGDAwz5CH06wfgYGw==&uniplatform=NZKPT&language=CHS

[ref50] Mollen S. Ruiter R. A. Kok G. (2010). Current issues and new directions in psychology and health: what are the oughts? The adverse effects of using social norms in health communication. Psychology & Health. 25, 265–270. doi: 10.1080/0887044090326281 20391219

[ref51] Newman P. A. Logie C. H. Doukas N. Asakura K. (2013). HPV vaccine acceptability among men: a systematic review and meta-analysis. Sex. Transm. Infect. 89, 568–574. doi: 10.1136/sextrans-2012-050980, PMID: 23828943 PMC3812849

[ref52] Okuhara T. Okada H. Goto E. Tsunezumi A. Kagawa Y. Kiuchi T. (2022). Encouraging HPV vaccination via an evolutionary theoretical approach: a randomized controlled study in Japan. Vaccine 10:701. doi: 10.3390/vaccines10050701, PMID: 35632459 PMC9143842

[ref53] Okunade K. S. Sunmonu O. Osanyin G. E. Oluwole A. A. (2017). Knowledge and acceptability of human papillomavirus vaccination among women attending the gynaecological outpatient clinics of a university teaching hospital in Lagos Nigeria. J. Trop. Med. 2017, 1–6. doi: 10.1155/2017/8586459, PMID: 29410683 PMC5749286

[ref54] Pan X.-F. Li R. Pan A. Larson H. (2016). Human papillomavirus vaccine approval in China: a major step forward but challenges ahead. Lancet Infect. Dis. 16, 1322–1323. doi: 10.1016/S1473-3099(16)30450-9, PMID: 27998583

[ref55] Pask E. B. Rawlins S. T. (2016). Men’s intentions to engage in behaviors to protect against human papillomavirus (HPV): testing the risk perception attitude framework. Health Commun. 31, 139–149. doi: 10.1080/10410236.2014.940670, PMID: 26098812

[ref56] Patel C. Brotherton J. M. Pillsbury A. Jayasinghe S. Donovan B. Macartney K. . (2018). The impact of 10 years of human papillomavirus (HPV) vaccination in Australia: what additional disease burden will a nonavalent vaccine prevent? Eurosurveillance 23:1700737. doi: 10.2807/1560-7917.ES.2018.23.41.1700737, PMID: 30326995 PMC6194907

[ref57] Pérez S. Fedoruk C. Shapiro G. K. Rosberger Z. (2016). Giving boys a shot: the HPV vaccine’s portrayal in Canadian newspapers. Health Commun. 31, 1527–1538. doi: 10.1080/10410236.2015.1089466, PMID: 27123533

[ref58] Peterson L. M. Orr J. A. Rogelberg S. D. Olsen N. (2022). Social–contextual factors interact with masculinity to influence college men’s HPV vaccination intentions: the role of descriptive norms, prototypes, and physician gender. J. Behav. Med. 45, 825–840. doi: 10.1007/s10865-022-00350-1, PMID: 36066688 PMC9446639

[ref59] Petty R. E. Cacioppo J. T. (1984). The elaboration likelihood model of persuasion. Advances in Experimental Social Psychology. 19, 123–205. doi: 10.1016/S0065-2601(08)60214-2

[ref60] Prestwich A. Webb T. L. Conner M. (2015). Using theory to develop and test interventions to promote changes in health behaviour: evidence, issues, and recommendations. Curr. Opin. Psychol. 5, 1–5. doi: 10.1016/j.copsyc.2015.02.011

[ref61] Razai M. S. Oakeshott P. Esmail A. Wiysonge C. S. Viswanath K. Mills M. C. (2021). COVID-19 vaccine hesitancy: the five Cs to tackle behavioural and sociodemographic factors. J. R. Soc. Med. 114, 295–298. doi: 10.1177/01410768211018951, PMID: 34077688 PMC8209756

[ref62] Rimal R. N. Real K. (2003). Understanding the influence of perceived norms on behaviors. Commun. Theory 13, 184–203. doi: 10.1111/j.1468-2885.2003.tb00288.x

[ref63] Rogers R. W. (1975). A protection motivation theory of fear appeals and attitude change1. J. Psychol. 91, 93–114. doi: 10.1080/00223980.1975.9915803, PMID: 28136248

[ref64] Schmid P. Rauber D. Betsch C. Lidolt G. Denker M.-L. (2017). Barriers of influenza vaccination intention and behavior–a systematic review of influenza vaccine hesitancy, 2005–2016. PLoS One 12:e0170550. doi: 10.1371/journal.pone.0170550, PMID: 28125629 PMC5268454

[ref65] Singh D. Vignat J. Lorenzoni V. Eslahi M. Ginsburg O. Lauby-Secretan B. . (2023). Global estimates of incidence and mortality of cervical cancer in 2020: a baseline analysis of the WHO global cervical Cancer elimination initiative. Lancet Glob. Health 11, e197–e206. doi: 10.1016/S2214-109X(22)00501-0, PMID: 36528031 PMC9848409

[ref66] Staadegaard L. Rönn M. M. Soni N. Bellerose M. E. Bloem P. Brisson M. . (2022). Immunogenicity, safety, and efficacy of the HPV vaccines among people living with HIV: a systematic review and meta-analysis. Eclinicalmedicine 52, 1–19. doi: 10.1016/j.eclinm.2022.101585, PMID: 35936024 PMC9350866

[ref67] Stout M. E. Christy S. M. Winger J. G. Vadaparampil S. T. Mosher C. E. (2020). Self-efficacy and HPV vaccine attitudes mediate the relationship between social norms and intentions to receive the HPV vaccine among college students. J. Community Health 45, 1187–1195. doi: 10.1007/s10900-020-00837-5, PMID: 32418009 PMC7606315

[ref68] Tong K. K. He M. Wu A. M. Dang L. Chen J. H. (2021). Cognitive factors influencing COVID-19 vaccination intentions: an application of the protection motivation theory using a probability community sample. Vaccine 9:1170. doi: 10.3390/vaccines9101170, PMID: 34696278 PMC8537765

[ref69] Varer Akpinar C. Alanya Tosun S. (2023). Knowledge and perceptions regarding human papillomavirus (HPV) and willingness to receive HPV vaccination among university students in a north-eastern city in Turkey. BMC Womens Health 23:299. doi: 10.1186/s12905-023-02455-4, PMID: 37280608 PMC10243033

[ref70] Vorpahl M. M. Yang J. Z. (2018). Who is to blame? Framing HPV to influence vaccination intentions among college students. Health Commun. 33, 620–627. doi: 10.1080/10410236.2017.1289436, PMID: 28281783

[ref71] Wang G. S. (2021). Analysis and prospects of vaccine launch in China. Chin. J. Vaccines Immun. 27:116. doi: 10.19914/j.CJVI.2021016

[ref72] Wang L. D.-L. Lam W. W. T. Fielding R. (2017). Determinants of human papillomavirus vaccination uptake among adolescent girls: a theory-based longitudinal study among Hong Kong Chinese parents. Prev. Med. 102, 24–30. doi: 10.1016/j.ypmed.2017.06.021, PMID: 28652087

[ref73] Wang Y. Wei Y. Zhang S. (2023). Influence of descriptive norms on influenza vaccination intention: the mediating role of risk perception and moderating role of reference groups. Public Adm. Rev. 5, 138–161.

[ref74] Werner J. S. J. James W. T. (2000). Communication theories: Origins, methods and uses in the mass media: Huaxia Publishing House.

[ref75] World Health Organization (2017). Human papillomavirus vaccines: WHO position paper, May 2017–recommendations. Vaccine 35, 5753–5755. doi: 10.1016/j.vaccine.2017.05.06 28596091

[ref76] World Health Organization. (2024). Human papillomavirus (HPV) and cancer. Available online at: https://www.who.int/news-room/fact-sheets/detail/human-papillomavirus-(hpv)-and-cancer

[ref77] Wu M. (2022). What drives people to share misinformation on social media during the COVID-19 pandemic: a stimulus-organism-response perspective. Int. J. Environ. Res. Public Health 19:11752. doi: 10.3390/ijerph191811752, PMID: 36142031 PMC9517463

[ref78] Xiao X. Borah P. (2021). Do norms matter? Examining norm-based messages in HPV vaccination promotion. Health Commun. 36, 1476–1484. doi: 10.1080/10410236.2020.1770506, PMID: 32452218

[ref79] Xie L. Ren J. Min S. Zhu X. Xu D. Qiao K. . (2023). Knowledge, attitude, and perception regarding HPV-related diseases and vaccination among the general public in Guizhou Province of China. Vaccine 41, 1119–1131. doi: 10.1016/j.vaccine.2022.12.027, PMID: 36610933

[ref80] Yao P.-Y. Lin C.-Y. Ko N.-Y. Zou H. Lee C.-W. Strong C. (2022). Predicting human papillomavirus vaccine uptake in men who have sex with men the influence of vaccine price and receiving an HPV diagnosis. BMC Public Health 22:28. doi: 10.1186/s12889-021-12396-y, PMID: 34991553 PMC8740414

[ref81] Yarmohammadi S. Mousavi S. Ajori L. Mohammadzade F. Marashi T. (2023). Impact of attitude, subjective norms and perceived behavioral control on health behaviors among women with low-risk HPV, and the mediating role of behavioral intention: an interventional study. Int. J. Health Promot. Educ. 61, 70–82. doi: 10.1080/14635240.2021.1995776

[ref82] Zhang H. Du J. Wang R. (2019). Media credibility: the impact of privately-owned websites on state-owned televisions in the context of China. J Asian Pacif. Commun. 29, 188–210. doi: 10.1075/japc.00030.zha

[ref83] Zhang S. Grant L. H. Geipel J. Cui Z. Keysar B. (2023). The impact of informational intervention on HPV vaccination intention among heterosexual men. Vaccine 11:1653. doi: 10.3390/vaccines11111653, PMID: 38005985 PMC10674571

[ref84] Zhang Z. Shi J. Zhang X. Guo X. Yu W. (2023). Willingness of parents of 9-to-18-year-old females in China to vaccinate their daughters with HPV vaccine. Vaccine 41, 130–135. doi: 10.1016/j.vaccine.2022.11.016, PMID: 36411136

[ref85] Zhang M. Wei W. Li Q. Chen X. Zhang M. Zuo D. . (2021). Determinants of intention to participate in breast cancer screening among urban Chinese women: an application of the protection motivation theory. Int. J. Environ. Res. Public Health 18:11093. doi: 10.3390/ijerph182111093, PMID: 34769613 PMC8583142

[ref86] Zhao X. Lynch J. G. Jr. Chen Q. (2010). Reconsidering baron and Kenny: myths and truths about mediation analysis. J. Consum. Res. 37, 197–206. doi: 10.1086/651257

[ref87] Zhao E. Wu Q. Crimmins E. M. Ailshire J. A. (2020). Media trust and infection mitigating behaviours during the COVID-19 pandemic in the USA. BMJ Glob. Health 5:e003323. doi: 10.1136/bmjgh-2020-003323, PMID: 33037063 PMC7545496

[ref88] Zhou X. Gao H. Wang J. (2025). A computational framework analysis of public attitudes toward male human papillomavirus infection and its vaccination in China: based on Weibo data. Healthcare 13, 1–17. doi: 10.3390/healthcare13030287, PMID: 39942476 PMC11818045

[ref89] Zhou Y. Zhang A. Liu X. Tan X. Miao R. Zhang Y. . (2023). Protecting public’s wellbeing against COVID-19 infodemic: the role of trust in information sources and rapid dissemination and transparency of information over time. Front. Public Health 11:1142230. doi: 10.3389/fpubh.2023.1142230, PMID: 37139363 PMC10149692

[ref90] Zou K. Huang Y. Li Z. (2022). Prevention and treatment of human papillomavirus in men benefits both men and women. Front. Cell. Infect. Microbiol. 12:1077651. doi: 10.3389/fcimb.2022.1077651, PMID: 36506029 PMC9729793

